# A Comparative Study on Visual Choice Reaction Time for Different Colors in Females

**DOI:** 10.1155/2014/301473

**Published:** 2014-12-16

**Authors:** Grrishma Balakrishnan, Gurunandan Uppinakudru, Gaur Girwar Singh, Shobith Bangera, Aswini Dutt Raghavendra, Dinesh Thangavel

**Affiliations:** ^1^Department of Physiology, Yenepoya Medical College, Yenepoya University, Deralakatte, Mangalore, Karnataka 575018, India; ^2^Department of Surgery, Yenepoya Medical College, Yenepoya University, Deralakatte, Mangalore, Karnataka 575018, India; ^3^Department of Physiology, Jawaharlal Institute of Post Graduate Medical Education and Research, Institute of National Importance, Ministry of Health and Family Welfare, Puducherry 605006, India; ^4^Department of Physiology, Dhanalakshmi Srinivasan Medical College, Perambalur, Tamil Nadu 621113, India

## Abstract

Reaction time is one of the important methods to study a person's central information processing speed and coordinated peripheral movement response. Visual choice reaction time is a type of reaction time and is very important for drivers, pilots, security guards, and so forth. Previous studies were mainly on simple reaction time and there are very few studies on visual choice reaction time. The aim of our study was to compare the visual choice reaction time for red, green, and yellow colors of 60 healthy undergraduate female volunteers. After giving adequate practice, visual choice reaction time was recorded for red, green, and yellow colors using reaction time machine (RTM 608, Medicaid, Chandigarh). Repeated measures of ANOVA and Bonferroni multiple comparison were used for analysis and *P* < 0.05 was considered statistically significant. The results showed that both red and green had significantly less choice visual choice reaction (*P* values <0.0001 and 0.0002) when compared with yellow. This could be because individual color mental processing time for yellow color is more than red and green.

## 1. Introduction 

Reaction is a purposeful voluntary response to an external stimulus. There is certain time period between application of external stimulus and appropriate motor response to the stimulus called the reaction time. Reaction time is defined as interval of time between presentation of stimulus and appearance of appropriate voluntary response in a subject [1, 2]. It is usually expressed in milliseconds. It reflects the speed of the flow of neurophysiological, cognitive, and information processes which are created by the action of stimulus on the person's sensory system. The receipt of information (visual or auditory), its processing, decision making, and giving the response or execution of the motor act are the processes which follow one another and make what we call the reaction time [3–5].

Concept of the reaction time of man appeared in science in the forties of the last century. Hermann von Helmholtz worked on nerve conduction velocity, a component of reaction time. He stimulated first one point of the nerve near to the muscle and then another point far from the muscle. The difference between the times from the stimulation of nerve to the muscle contraction in those two situations is the nerve conduction velocity. Later experiments were done to study the time taken for a specific response which was called reaction time [6]. Reaction time is very important for our everyday lives and needs intact sensory system, cognitive processing, and motor performance. Reaction time is a good indicator of sensorimotor coordination and performance of an individual. Reaction time determines the alertness of a person and must be lesser in certain occupations, for example, drivers, military people, pilots, sportsmen, doctors, nursing staff, and security guards where alertness is a must for them [1].

Many factors have been shown to affect reaction time including gender, age, physical fitness, level of fatigue, distraction, alcohol, personality type, limb used for test, biological rhythm, and health and whether the stimulus is auditory or visual [5]. Reaction time is independent of social-cultural influences. Prolonged reaction time denotes decreased performance [7].

There are 3 different types of reaction time experiments, simple, recognition, and reaction time experiments. In simple reaction time experiments, there is only one stimulus and one response. In recognition reaction time experiments, there are some stimuli (the “memory set”) that should be responded to and others (the “distracter set”) that should not be responded to. In choice reaction time experiments, there are multiple stimuli and multiple responses and subject must give a response that corresponds to the stimulus [8]. It was reported that the time for motor preparation (e.g., tensing muscles) and motor response was the same in all three types of reaction time tests, implying that the differences in reaction time are due to processing time [5, 8].

Many studies have been undertaken to examine the influence of color on the simple reaction time. In few, reaction time has been shown to be independent of wavelength while others have found that reaction time to red stimuli was shorter than that to green or blue stimuli [9–12]. The issue of variation in RT with changing stimulus chromaticity therefore merits reexamination. Also, in everyday life choice reaction time becomes more important than the simple reaction time.

The choice reaction time can be studied by using visual inputs or by using auditory inputs. When studied using visual inputs it is called visual choice reaction time. Contemporary models of color vision assume that chromatic information is extracted through two independent postreceptoral cone-opponency channels, processing red-green (L-M) and blue-yellow (S-[L-M]) information (where S, M, and L represent input from short, middle, and long wavelength sensitive cones, resp.) [11]. Because of this, red, green, and yellow colors were used for the study. Reaction time is faster when the dominant hand is used when compared with the opposite side. Visual choice reaction time using the dominant limbs was studied. Reaction time is faster in men compared with women [13]. For uniformity, we had analyzed the visual choice reaction time on 60 female subjects.

*Aim of the Study.* The aim is to compare the visual choice reaction time for red, green, and yellow colors of 60 healthy undergraduate female subjects.

## 2. Materials and Methods

The study was conducted in Department of Physiology, Jawaharlal Institute of Post Graduate Medical Education and Research (JIPMER), Pondicherry, India. Prior to commencement of study approval of JIPMER scientific advisory committee and ethics committee was obtained. Sixty healthy female volunteers without visual defects or with corrected (with glasses) visual defects were recruited for the study. Visual choice reaction time for red, green, and yellow colors was compared. All tests were carried out in Autonomic Function Testing Laboratory in the Department of Physiology, JIPMER, between 3.00 pm and 5.00 pm. The laboratory environment was quite and the temperature was maintained between 22 and 25°C. The participants were informed in detail about study protocol and written informed consent was obtained from them. The subjects were advised to have lunch at 1.00 pm and come for tests at least two hours after lunch with empty bowel and bladder. The subjects were instructed to avoid caffeine and nicotine 12 hours before and sympathomimetics and parasympathomimetic agents, psychotropic drugs (sedatives, hypnotics, and tranquilizers), and antihistamines 48 hours prior to the study. The parameters were recorded 6–8 days after menstruation. The anthropometric measurements were taken. Subject's height was measured to the nearest millimeter by a wall mounted stadiometer. Weight was measured with an electronic weighing scale (Microgene, New Delhi) with LCD with accuracy of ±0.1 kg. BMI was calculated by Quetelet's index that is weight/[height]^2^, weight in kg and height in meters. Visual reaction time was done in subjects using reaction time machine (RTM-608, Medicaid Systems, Chandigarh) with resolution of 0.001 sec, accuracy ±1 digit, and 3 different lights, red, green, and yellow and 3 different sounds, high, medium, and low pitch sounds.

The subjects were instructed about the procedure and after adequate practice the tests were performed. Each of the three lights, namely, yellow, red and green lights, had its corresponding button equidistant from centre button. Keeping the same luminance for all three colors, we studied the reaction response to changes only in chromaticity. The subject was asked to keep the index finger of the dominant hand on the center button and press the corresponding light button as soon as yellow, red, or green light appears. The reaction values were directly read from digital display.

### 2.1. Statistical Analysis

Ten values of visual reaction time were recorded, two lowest and two highest values were deleted, and the average for the middle six values was calculated. The data were summarized using descriptive statistics, mean and standard deviation. Repeated measures of ANOVA and Bonferroni multiple comparison were used for analysis using appropriate statistical software. *P* < 0.05 was considered statistically significant.

## 3. Results

**Figure 1:**
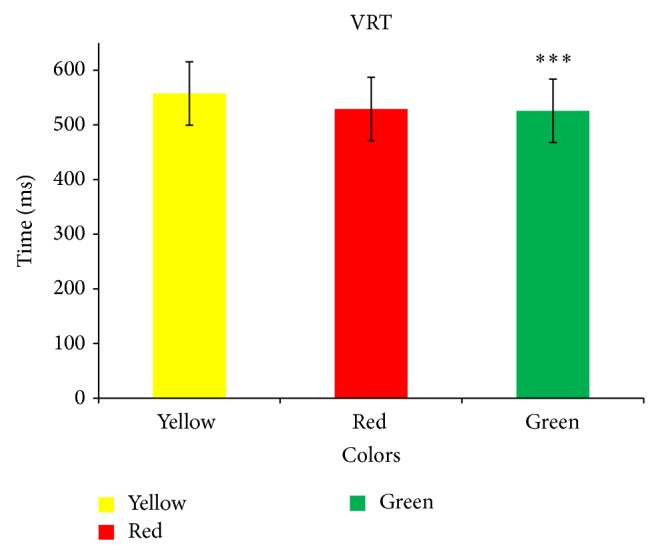
Comparison of visual choice reaction time among colors. Values are expressed as mean ± SD; analysis was done by repeated measures ANOVA. ^*^ *P* < 0.05; ^**^ *P* < 0.01; ^***^ *P* < 0.001. *F* value, 15.01. VRT: visual reaction time.

Table 1 shows mean age group and anthropometric measurements of sixty study subjects. When the visual choice reaction time among the colors was compared using repeated measures ANOVA, it showed visual choice reaction time in milliseconds for yellow, red, and green was statistically significant with *P* value <0.001 and *F* value 15.01 (Figure 1). Visual choice reaction time among colors was compared in pairs using Bonferroni multiple comparison test and it showed that both red and green color choice visual reaction times were significantly less when compared with yellow with *P* values <0.0001 and 0.0002, respectively (Table 2).

## 4. Discussion

Reaction time is one of the important methods to study a person's central information processing speed and coordinated peripheral movement response [1]. Cognitive processes are typically inferred from behavioral data such as accuracy and reaction time [14]. Choice reaction time is very important in driving vehicles. Most of the time people drive their vehicles based on the conditioned reflexes, learned through experience, but sometimes when unexpected situation arises, like when they suddenly spot a traffic signal, their reaction to it is an example of visual choice reaction time. Henry and Rogers proposed “memory drum” theory according to which complex responses, like responses for choice reaction, required more stored information and hence take longer time to react [15].

The results obtained by different authors show that when a color stimulus changes in both luminance and chromaticity, the visual reaction time of an observer in detecting this chromatic change depends on nothing more than the luminance change and is regulated by Pieron's law [16]. The purpose of our study was to compare the visual choice reaction time for red, green, and yellow colors keeping the luminance constant in 60 healthy undergraduate female subjects. We had included only females in our study because reaction time is known to be faster in men compared with women [13]. The findings of our study revealed both red and green color choice visual reaction times were significantly less when compared with yellow. Our findings are consistent with Venkatesh et al. who had reported that green color evoked a faster response due to its stronger stimulation on the visual receptors and refute the findings of study conducted by Hita et al. who reported that there was no correlation between reaction time and chromaticity [17, 18]. But both their studies were on simple reaction time.

The components of choice reaction time are (1) mental processing time, (2) nerve conduction time, (3) movement time (including motor preparation and motor response), and (4) device response time [19, 20]. Since the nerve conduction time, movement time, and device response time are the same for all the three colors, the difference in visual choice reaction time should be in individual color mental processing time. Choice reaction time is also a function of stimulus information but only up to some amount of practice, after which it is independent of the number of alternatives; to minimize its influence on results we have used the same number of colors with adequate practice [21].

A three-state conceptualization of the central mechanisms or mental processing time operative during the latent period-preprocessing sensation (the time it takes to detect the sensory input from an object), stimulus categorization (according to Donders it includes stimulus-stimulus translation and stimulus-response translation), and response selection is proposed. The stimulus detection could contribute to increased visual choice reaction time for yellow when compared to the other two colors, as red-green activates (L-M) cone and blue-yellow activates S-[L-M]. It is reported that simple RTs generated in response to S cone-isolating stimuli are longest, whereas the shortest RTs are generated by L-M cone-isolating stimuli [12, 21].

The difference in visual choice reaction time among colors with increased time for yellow color when compared with red and green colors could be because of difference in time taken for stimulus categorization and response selection. Stimulus categorization includes process-template matching versus feature testing [20]. Increased neuronal gamma-band synchronization and shortened neuronal response latencies to stimulus have direct effects on visually triggered behavior and reflect visuomotor integration. Hence we can say that gamma-band synchronization is better for red and green colors when compared with yellow color [22]. We could not separately measure the components of mental processing time to strengthen our findings. In addition, visual choice reaction time for shorter wave length light could also be recorded which forms the future scope of our study.

## 5. Conclusion

The study results indicated visual choice reaction time for yellow color was significantly more than red and green colors. This could be because individual color mental processing time for yellow color is more than red and green. The difference could be in either preprocessing sensation and stimulus, stimulus categorization, or response selection or all of them. Hence we suggest that yellow color and its variants should be less used in places where reaction time becomes very important like in traffic signals and so forth.

## Figures and Tables

**Table 1:** Age and anthropometric measurements of the subjects.

Parameters	Study group (*n* = 60)
Age (years)	19.23 ± 0.86
Weight (kg)	52.24 ± 9.09
Height (cms)	156.81 ± 4.37
BMI (kg/m^2^)	21.40 ± 3.57

Values are expressed as mean ± SD. BMI: body mass index.

**Table 2:** Pairwise comparison of visual choice reaction time among colors.

Pairs			*P* value
VRT green	—	VRT red	1.0000
	—	VRT yellow	0.0002

VRT red	—	VRT green	1.0000
	—	VRT yellow	<0.0001

VRT yellow	—	VRT green	0.0002
	—	VRT red	<0.0001

VRT yellow: visual reaction time to yellow light, VRT red: visual reaction time to red light, and VRT green: visual reaction time to green light.
